# Associations of *MEG3* rs7158663, rs4081134 gene variants and Ki-67, p53, CK18 immunohistochemical markers with clinical features of pituitary neuroendocrine tumors

**DOI:** 10.3389/fendo.2025.1657520

**Published:** 2025-10-09

**Authors:** Balys Remigijus Zaliunas, Enrika Pileckaite, Monika Duseikaite, Jurgita Makstiene, Lina Poskiene, Vita Rovite, Sheng-Nan Wu, Arimantas Tamasauskas, Rasa Liutkeviciene

**Affiliations:** ^1^ Medical Academy, Lithuanian University of Health Sciences, Kaunas, Lithuania; ^2^ Laboratory of Ophthalmology, Institute of Neuroscience, Lithuanian University of Health Sciences, Kaunas, Lithuania; ^3^ Department of Pathology, Lithuanian University of Health Sciences, Kaunas, Lithuania; ^4^ Latvian Biomedical Research and Study Centre, Riga, Latvia; ^5^ Department of Neurology, National Cheng Kung University Hospital, Tainan, Taiwan; ^6^ Department of Neurosurgery, Hospital of Lithuanian University of Health Sciences, Kaunas Clinics, Kaunas, Lithuania

**Keywords:** pituitary adenoma, p53, CK18, Ki-67, *MEG3*, PitNET, immunohistochemistry, biomarkers

## Abstract

**Aim:**

This study aimed to determine the associations of *MEG3* rs7158663, rs4081134 gene variants, as well as the immunohistochemical markers Ki-67, p53, and CK18, with the clinical features of pituitary neuroendocrine tumors (PitNETs).

**Methods:**

This case-control study included 340 individuals who were divided into two groups: a control group (*n=220*) and a PitNETgroup (*n=120*). DNA was isolated from the venous blood of study participants by the leukocyte salt precipitation method. Real-time polymerase chain reaction was used for the *MEG3* rs7158663, rs4081134 single nucleotide variants genotyping. Immunohistochemical analysis of Ki-67 labeling index and p53 protein biomarkers was performed using the automated Ventana BenchMark ULTRA PLUS staining system, following the manufacturer’s recommendations. CK18 immunostaining was conducted with the Dako Omnis staining system, following the manufacturer’s recommendations. Monoclonal antibodies SP6, DO-7, and DC-10 were used to detect Ki-67 labeling index, p53, and CK18, respectively. Statistical data analysis was performed using the “IBM SPSS Statistics 30.0” program.

**Results:**

Genotype and allele frequencies of *MEG3* rs7158663 and rs4081134 variants showed no significant differences between healthy controls and PitNET patient groups. Additionally, no associations were found between either *MEG3* variants and PitNET recurrence, size, invasiveness, and functional status. Ki-67 labeling index (>3% vs. ≤3%) showed no significant differences with any clinical feature of PitNETs (recurrence, size, invasiveness, functional status). In contrast, the p53 H-score was significantly higher in macroadenomas than in microadenomas (median 27 vs. 16; *p=*0.008). Additionally, invasive pituitary adenomas showed a higher p53 H-score compared with non-invasive tumors (median 27 vs. 20; *p=*0.018). Negative CK18 immunostaining was significantly more frequent in invasive than non-invasive PitNETs (44.4% vs. 13.3%; *p* < 0.001) and in non-functioning compared to functioning adenomas (42.0% vs. 18.4%; *p=*0.011). No significant associations were found between either *MEG3* variant and Ki-67 LI, p53 H-score, or CK18 immunohistochemical reactions.

**Conclusions:**

This study found that a higher p53 H-score was significantly associated with larger PitNET size and invasiveness. Negative CK18 staining was associated with non-functioning and invasive PitNETs. P53 expression and CK18 status may serve as useful prognostic markers in PitNETs.

## 1 Introduction

Pituitary neuroendocrine tumors (PitNETs) are the most common sellar-region tumors, classified as functioning or nonfunctioning depending on their hormonal activity. According to data from studies in the general population, the prevalence of clinically significant PitNETs is around 1 in 1100 people. Interestingly, prolactinomas account for approximately 53% of PitNETs, and occur about ten times more frequently in women than in men (1). Depending on their size, PitNETs are classified as microadenomas (<1 cm) or macrodenomas (>1 cm). Macroadenomas often cause the compression of the optic chiasm, leading to visual impairment (bitemporal hemianopsia) (2). In addition, macroadenomas can also present with headaches or hypopituitarism due to the mass effect (3). Based on their hormonal activity, functioning PitNETs are classified by lineage into gonadotroph, thyrotroph, corticotroph, lactotroph, and somatotroph tumors, which clinically present as hypogonadism, hyperthyroidism, Cushing’s disease, hyperprolactinemia, and acromegaly, respectively (4). On the other hand, clinically nonfunctioning PitNETs are usually associated with mass-effect symptoms and hypopituitarism (5).

Based on the 2022 World Health Organization (WHO) Classification of Endocrine and Neuroendocrine Tumors, PitNETs are grouped by cellular lineage, which is determined through targeted immunohistochemical reactions. PitNETs of PIT1 lineage includes somatotroph, lactotroph, and thyrotroph adenomas. Furthermore, corticotroph and gonadotroph adenomas and their variants comprise TPIT and SF1 lineages, whereas tumors that lack detectable hormone or transcription factor expression are defined as null-cell adenomas (6, 7). However, to date, no distinctive morphologic criteria and no single prognostic biomarker can predict the likelihood of tumor growth or malignant progression (4, 8, 9).

Maternally expressed gene 3 (*MEG3*) is a long non-coding RNA (lncRNA) located on chromosome 14q32.3. One of the main functions of *MEG3* lncRNA is the regulation of the p53 tumor suppressor gene expression and the induction of p53-dependent transcription through multiple mechanisms (10). By interacting with the polycomb repressive complex 2 (PRC2) and its cofactor Jumonji, and AT-rich interaction domain-containing 2 (JARID2), *MEG3* inhibits the expression of MDM2 and CDH1, which leads to p53 activation and a reduction in epithelial-mesenchymal transition (EMT), respectively. In addition, *MEG3* can directly bind to p53 and activate the expression of growth differentiation factor 15 (GDF15). Through these interactions, *MEG3* inhibits the proliferation and invasion of cancer cells (11–13). *MEG3* has been found to be downregulated in a wide range of neoplasms, including squamous cell carcinoma of the head and neck, neuroblastoma, glioma, meningioma, retinoblastoma, and thyroid cancer. However, relatively few studies have investigated the relationship between *MEG3* lncRNA and PitNETs (14).

Ki-67 is a nuclear protein associated with cellular proliferation and is detected in all active phases of the cell cycle (G_1_, S, G_2_, and M) except G_0_. Encoded by the human *MKI67* gene, Ki-67 plays multiple roles during mitosis, including the assembly of the perichromosomal protein compartment, the organization of heterochromatin, the attachment of chromosomes to the mitotic spindle, and the exclusion of large cytoplasmic molecules from the nuclei at the end of mitosis (15). Numerous studies have demonstrated that a higher Ki-67 labeling index (LI) is associated with poorer survival, larger tumor size, lymphatic invasion, and metastasis (16). In the context of neuroendocrine tumors, several studies have evaluated the prognostic role of the Ki-67 LI. Combined with morphological and radiological evidence, Ki-67 LI shows promising signs for identifying PitNETs at higher risk of recurrence or local invasion (17, 18). Accordingly, immature PIT1-lineage, Crooke cell, null-cell, silent corticotroph, sparsely granulated somatotroph, and corticotroph adenomas demonstrate more aggressive behavior (19–22). However, the independent prognostic role of Ki-67 in PitNETs remains unclear.

p53 is a transcription factor that is inactivated in nearly all tumors, and approximately 50% of these cases are associated with mutations in the *TP53* (23). In addition to its roles in cell-cycle arrest, apoptosis, and senescence, p53 is responsible for a wide range of tumor-suppressive functions. p53 acts as a metabolic regulator that suppresses anabolic reactions (glycolysis, lipogenesis) and activates catabolic processes (oxidative phosphorylation, fatty acid oxidation). Additionally, p53 promotes ferroptosis, which serves as an alternative to apoptosis in eliminating cells under metabolic stress (24). While p53 functional role and immunohistochemical patterns have been widely studied across a range of cancers, including high‐grade serous carcinoma, endometrial carcinoma, high-grade bladder cancer, and prostate cancer, its specific involvement in PitNETs remains poorly understood (25–27).

Cytokeratin 18 (CK18) is an intermediate filament protein that is co-expressed with cytokeratin 8 (CK8), forming heteropolymers that assemble into keratin filaments, which are found in the cytoplasm of epithelial cells (28). Besides its structural and protective roles, CK18 is involved in multiple cellular processes, including apoptosis, cell cycle progression, mitosis, and cell signaling (29). A large study conducted by Menz and co-authors revealed the involvement of CK18 in various types of cancers. The study demonstrated that CK18 is strongly expressed in most adenocarcinomas, especially those originating from the lung, prostate, colon, pancreas, and ovary. In addition, tumors that arise from CK18-positive tissues, such as breast and renal cancers, may downregulate CK18 expression, leading to more aggressive tumor development and poorer prognosis (30). In the context of neuroendocrine tumors, CK18 immunohistochemistry is widely used for the histopathological classification of PitNETs. For instance, CK18 fibrous bodies in sparsely granulated somatotroph tumors, and Crooke’s cells in corticotroph adenomas (7, 31). However, to date, the relationships between CK18 immunohistochemical reaction types and clinical characteristics of PitNETs have not been established. Collectively, *MEG3*, Ki-67, p53 and CK18 provide unique insights into tumor biology. However, their impact on the clinicopathological characteristics of PitNETs remains unclear.

Given their involvement in various malignancies, this study aimed to investigate the clinicopathological roles of *MEG3* genetic variants, Ki-67, p53, and CK18 in the development of PitNETs, with the goal of clarifying their prognostic value, associations with tumor aggressiveness, and clinical characteristics, which may contribute to improved management of neuroendocrine tumors.

## 2 Research methodology

### 2.1 Organization of the research

This research was carried out in the Ophthalmology Laboratory of the Institute of Neurosciences of the Lithuanian University of Health Sciences (LUHS). DNA and tumor tissue samples were collected at the institution between 2014 and 2024 as part of routine diagnostic and research procedures. Kaunas Regional Biomedical Research Ethics Committee approved the study (No. BE-2-47, issued on 25 December 2016). The study was divided into four stages. In the first stage, an analysis of the scientific literature was performed, and DNA extraction was performed using the salting out method from the subjects’ venous blood leukocytes. In the second stage, the lncRNA *MEG3* rs4081134 and rs7158663 genotypes were determined using the real-time polymerase chain reaction (RT-PCR). In the third stage, immunohistochemical markers were determined: Ki67, p53, and CK18 at the Department of Pathological Anatomy of the LUHS hospital KK. In the fourth stage, the results were analyzed using the statistical program, the results were described, and a report was prepared.

### 2.2 Subject selection

The study participants were divided into two groups:

Patients with PitNET (n=120 patients): 69 women (57.5%) and 51 men (42.5%).

Patients who were 18 years of age or older, in good general health, and free of any tumors (except pituitary neoplasms) were included in the PitNET group. PitNETs were identified and verified by magnetic resonance imaging (MRI).

The PitNET group was split up into smaller groups based on relapse, hormonal activity, and invasiveness. Groups were separated into invasive and non-invasive categories, based on histopathological views; active and inactive PitNET groups were formed based on blood serum hormone levels. If a follow-up MRI revealed a new growth or the expansion of a remaining tumor, relapse was indicated. When a new growth or expansion of a residual tumor was seen on follow-up MRI following surgical excision during the research period, PitNET recurrence was identified. If a follow-up MRI showed no indications of tumor progression, the remaining tumor was deemed stable.

A control group consisting of 220 individuals: 120 women (54.5%) and 100 men (45.5%).

The participants in the healthy control group were matched by age and gender to those in the PitNET group and were generally in good health.

All study subjects signed an informed consent form to participate in this study. Blood was collected from the peripheral vein of each study participant.

### 2.3 DNA extraction and genotyping

Blood from all study participants was collected in vacuum tubes containing ethylenediaminetetraacetate (EDTA) to prevent clot formation. In this study, DNA was isolated from the venous blood of study participants using the leukocyte salt precipitation method. The method was used as described earlier (32). The lncRNA genotypes of the *MEG3* rs4081134 and rs7158663 variants were determined by RT-PCR using the StepOne Plus amplifier (Applied Biosystems, USA). The genotypes of selected lncRNA variants were determined according to the protocols of the manufacturer, StepOne Plus (Applied Biosystems). The protocol for determining genotyping conditions and variant characteristics is presented in **Table 1**.

**Table 1:** Genotype determination protocol and lncRNA variants information.

Variants	Assay id	Manufacturer	RT-PCR conditions
*MEG3* rs4081134	C:_1259786_10	TaqMan^®^ Genotyping assays (Applied Biosystems, New York, NY, USA; Thermo Fisher Scientific, Inc., Waltham, MA, USA)	95°C 10 minutes. 45 cycles 92°C 15 sec. 60°C 60 sec.
*MEG3* rs7158663	C:_9693465_10		

### 2.4 SNV selection

Both of our chosen SNVs (rs7158663 and rs4081134) are intronic; such variants can have a significant impact on lncRNA expression, splicing, or structure, which may in turn influence downstream tumor suppressor pathways. In lncRNAs like *MEG3*, intronic regions may also contain regulatory motifs or binding sites essential for RNA stability or interaction with proteins and other RNAs (10). These SNVs have previously studied cancer for susceptibility and prognosis in other populations. In particular, rs7158663 has been reported to affect *MEG3* expression levels, possibly influencing cancer risk (14). To our knowledge, rs7158663 and rs4081134 have not yet been thoroughly investigated in the context of PitNETs, which provided an opportunity to explore novel associations and contribute new data to the field.

### 2.5 Immunohistochemistry

After fixation of the surgical material with 10% formalin solution, standardized paraffin embedding procedures of PitNET tissues were performed using the Shandon Pathcentre processor (Shandon, UK). After forming paraffin blocks of PitNET tissues in the TBS88 system (Medite, Germany), 2 μm-thick sections were made using a Leica RM2125RT rotary microtome (Leica Biosystems, Germany).

Immunohistochemical reactions of Ki-67 LI and p53 protein biomarkers were performed using the automated Ventana BenchMark ULTRA PLUS staining system (Roche Diagnostics, Switzerland) according to the manufacturer’s recommendations. The Dako Omnis staining system (Agilent Technologies, CA, USA) was used for CK18 analysis according to the manufacturer’s recommendations. Ki-67 LI, p53, and CK18 were detected using monoclonal antibodies, the clones of which are SP6 (Vitro S.A., Spain), DO-7 (Roche Diagnostics, Switzerland), and DC-10 (Vitro S.A., Spain), respectively. After performing Ki-67, p53, and CK18 immunohistochemical reactions, the images were digitized with a Pannoramic 250 FLASH III scanner (3DHISTECH, Hungary). The evaluation of the digitized images for Ki-67, p53, and CK18 was performed by a qualified pathologist at the Clinic of Pathological Anatomy of the LUHS, using the 3DHISTECH SlideViewer 2.9.0. program and based on the WHO Classification of Endocrine and Neuroendocrine Tumors (6).

When evaluating CK18 images, immunohistochemical reactions were divided into positive (+) and negative (-). Diffuse and focal staining patterns were determined for positive reactions. Depending on the nature of the staining, cytoplasmic, perinuclear, fibrous body, and ring-like perinuclear reactions were observed (**Table 2**).

**Table 2:** Types of immunohistochemical reactions of PitNET with CK18.

Tumor type	Perinuclear reactions	
PIT1 lineage tumors: • Somatotrophic PitNET • Mammosomatotrophic PitNET • etc.	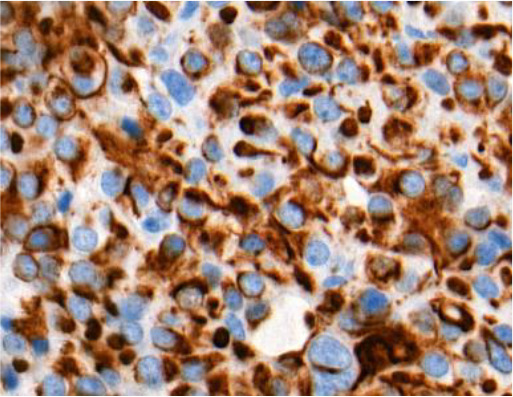	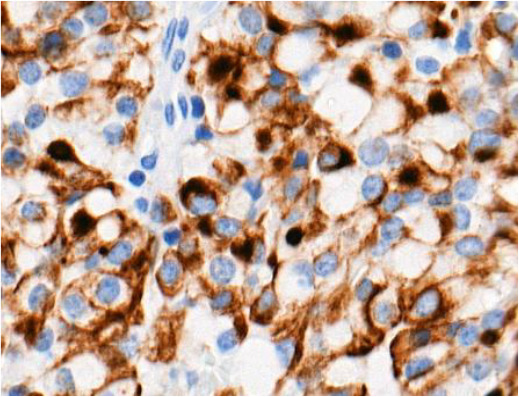

Tumor type	Ring-like perinuclear reactions	
TPIT lineage tumors: • Corticotrophic PitNET (Crooke adenoma)	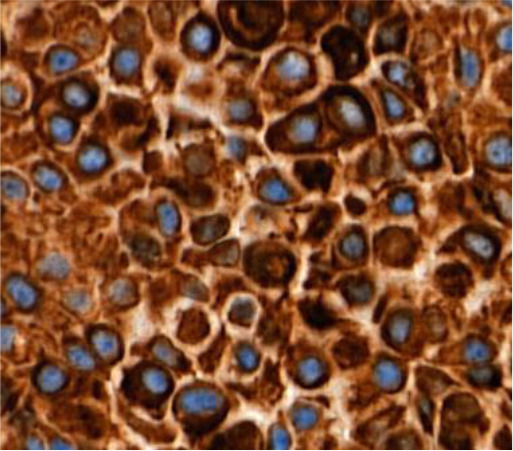	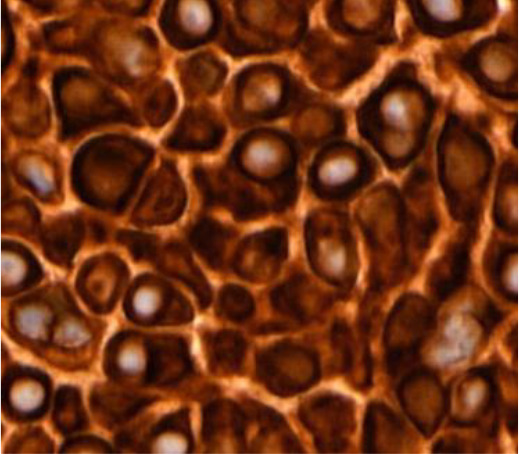

Tumor type	Fibrous body reactions	
PIT1 lineage tumors: • Somatotrophic PitNET • Acidophilic stem cells PitNET • etc.	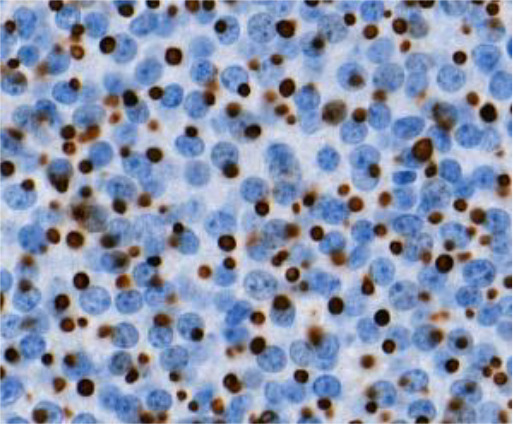	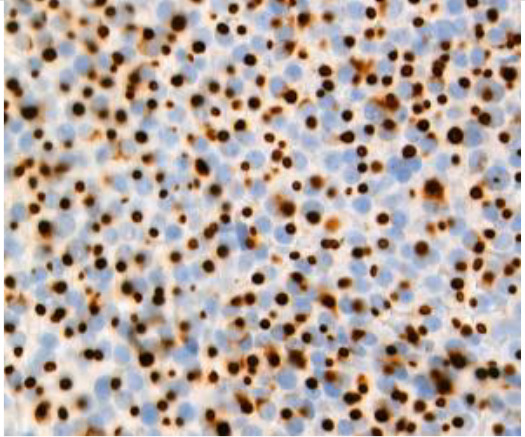

Ki-67 and p53 marker assessments were performed in hotspot zones - the most densely positively stained regions (**Table 3**). After selecting the hotspot zone with the best scanning quality, 300 tumor cell nuclei were evaluated, which falls below recommended standards. However, this approach ensured consistency and feasibility across all cases. When assessing Ki-67, cell nuclei were divided into “Negative (-)” and “Positive (+)” categories and the percentage of positively stained nuclei was calculated. When evaluating p53, tumor cell nuclei were divided into “Negative (-)”, “Weakly positive (+)”, “Moderately positive (++)”, and “Strongly positive (+++)” categories, taking into account the color of the nuclear staining, its intensity, and distribution throughout the nucleus. The percentage of all p53-positive nuclei was calculated and divided by category separately. Morphologically unclear nuclei were not included in the evaluation.

**Table 3:** Ki-67 and p53 evaluation in PitNET.

Ki-67 evaluation	
Before evaluation	After evaluation
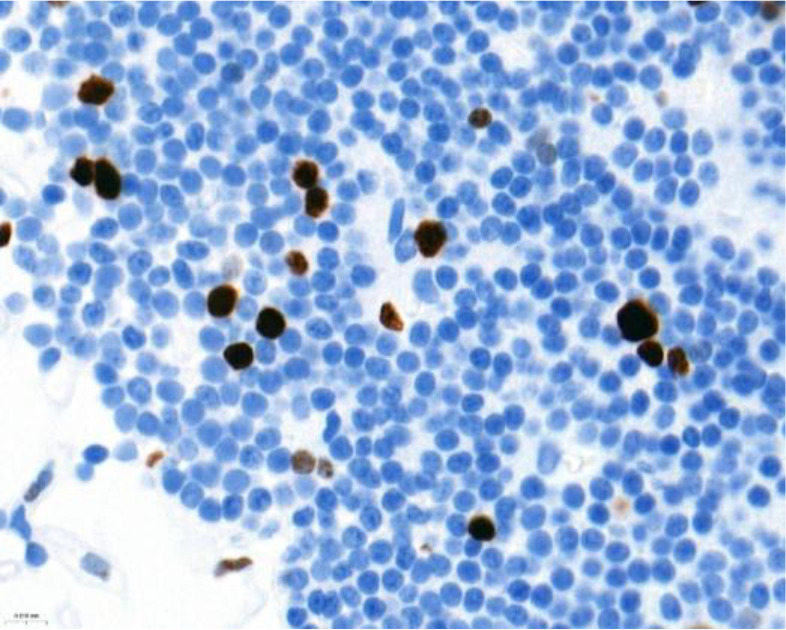	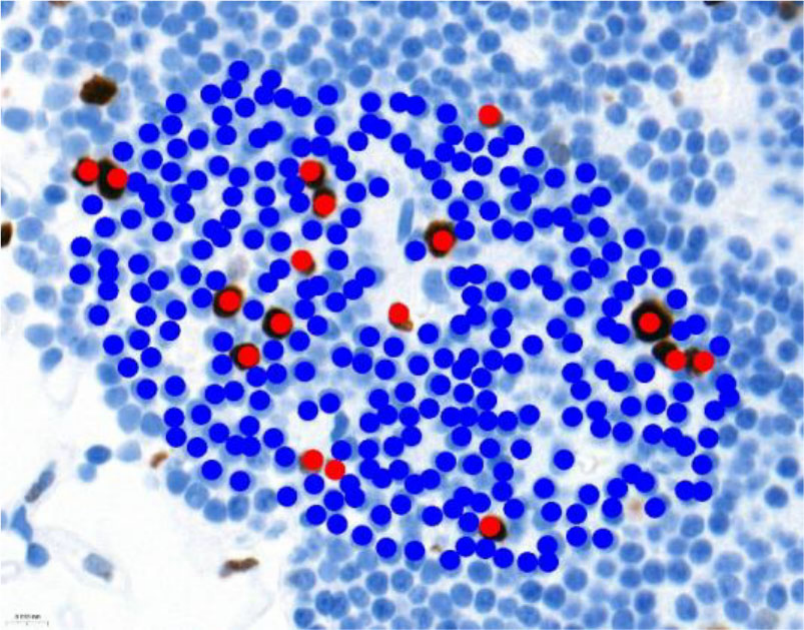

p53 evaluation	
Before evaluation	After evaluation
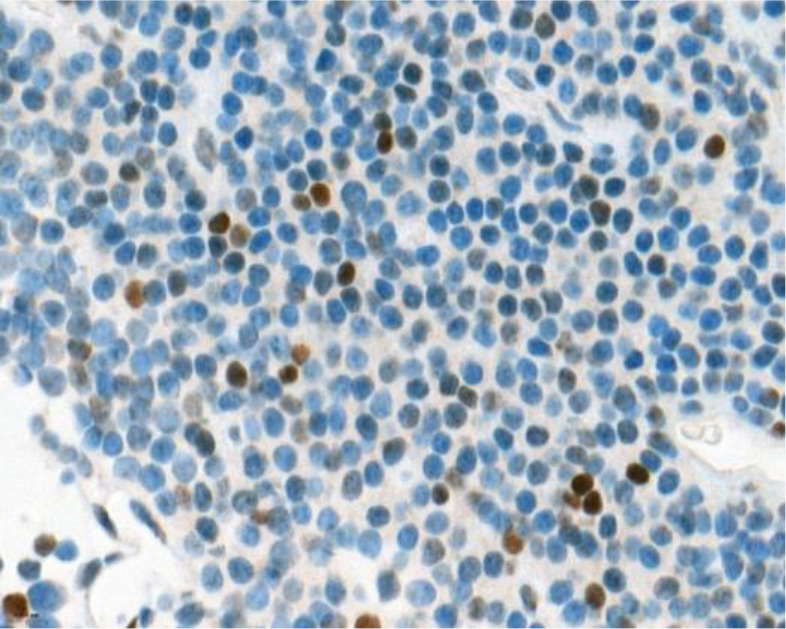	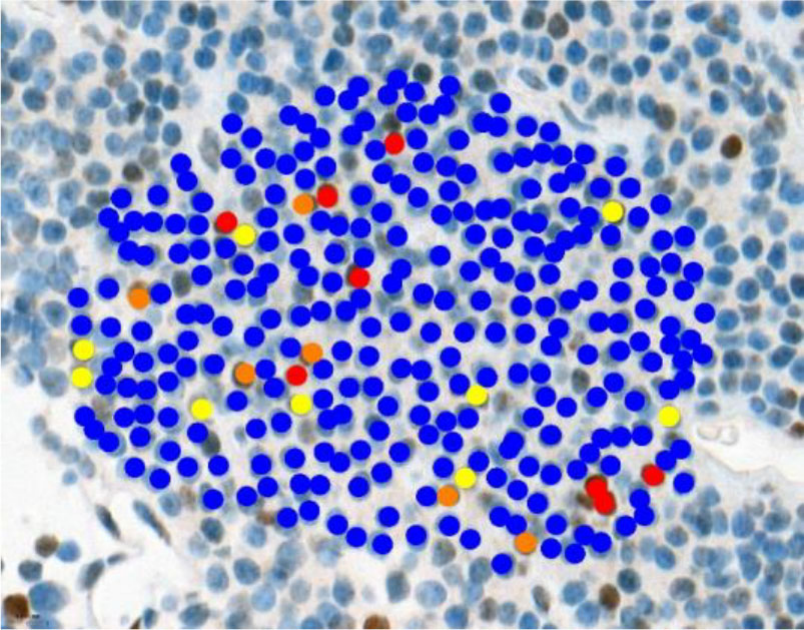

To calculate the percentage of Ki-67-positive (brown/dark brown/black) nuclei, negative nuclei were marked in blue and positive nuclei in red. For p53-positive nuclei, “weakly positive (+)” nuclei were marked in yellow, “moderately positive (++)” in orange, and “strongly positive (+++)” in red.

### 2.6 Statistical analysis

Using the global prevalence of pituitary adenomas of 9% (1) and the minor allele frequencies of *MEG3* rs4081134 (31.4%) and rs7158663 (46.9%) from the dbSNP database (33, 34), we calculated the statistical power for detecting associations under the most robust genetic models selected via the lowest AIC in logistic regression. With a sample size of 120 PitNET patients and 220 controls, the study achieved less than 80% power for detecting moderate genetic effects, indicating that future research with larger sample sizes is warranted to confirm these findings. Statistical analysis was performed using the Statistical Package for the Social Sciences, version 29.0 for Windows (SPSS for Windows, version 29.0, USA). The hypothesis of normal distribution for the measured variables was tested using the Kolmogorov-Smirnov test. When the data did not meet the criteria for normal distribution, descriptive statistics such as the median and interquartile range (IQR) were applied, with the non-parametric Mann-Whitney U test. The χ² test was used to analyze the differences in the distribution of single nucleotide variants in *MEG3* rs4081134 and rs7158663. Binary logistic regression analysis was used to estimate the odds ratio (OR) of disease occurrence, indicating the OR with a 95% confidence interval (CI). The Akaike Information Criterion (AIC) was used to determine the best-fitting inheritance model, with the lowest AIC value indicating the most appropriate model. According to the 2022 WHO Classification of Endocrine and Neuroendocrine Tumors (6), Ki-67 results were categorized into two groups: “≤3%” and “>3%”. For p53, tumor cell nuclei were classified as “Negative (-)”, “Weakly positive (+)”, “Moderately positive (++)”, or “Strongly positive (+++)”, based on nuclear staining color, intensity, and distribution. To objectively assess p53 staining intensity, a Histoscore (H-score) was calculated as follows: (1 × percentage of weakly staining nuclei) + (2 × percentage of moderately staining nuclei) + (3 × percentage of strongly staining nuclei). To test statistical hypotheses, we chose the significance level (*p*) criterion, the Bonferroni correction was applied to the SNV analysis, and a statistically significant difference was determined when the *p*-value was <0.025. In another analysis, statistically significant changes were those with *p* < 0.05.

## 3 Results

The case-control study involved 340 subjects divided into two groups: a control group (n=220) and a group of PitNET patients (n=120). After the subject groups were formed, genotyping of the *MEG3* rs4081134 and rs7158663 variants was performed. The PitNET group consisted of 120 subjects: 51 males (42.5%) and 69 females (57.5%). The control group consisted of 220 subjects: 100 males (45.5%) and 120 females (54.5%). The demographic data of the subjects are presented in **Table 4**.

**Table 4:** Characteristics of study subjects.

Characteristic		Group		*p-*Value
		PITNET(%)	Control (%)	
Gender	Male	51 (42.5)	100 (45.5)	0.600
	Female	69 (57.5)	120 (54.5)
Age median (IQR)		54 (20)	55 (21)	0.592

PitNET, pituitary adenoma; *p* – significance level when *p=*0.025; IQR – interquartile range *Pearson chi-square; **Mann-Whitney U test.

The genotypes and allele distributions of *MEG3* rs4081134 and rs7158663 were analyzed in the PitNET group and compared with the control group. However, no statistically significant differences were found between the groups (**Table 5**).

**Table 5:** *MEG3* rs4081134 and rs7158663 genotype and allele frequencies in PitNET and control groups.

Variant	PitNET group, N (%)	Control group, N (%)	*p-*Value	HWE *p-*Value
*MEG3* rs4081134 GG AG AA In total Allele G A	55 (45.8) 54 (45) 11 (9.2) 120 (100) 164 (68.3) 76 (31.7)	103 (46.8) 94 (42.7) 23 (10.5) 220 (100) 300 (68.2) 140 (31.8)	0.888 0.968	0.281
*MEG3* rs7158663 AA AG GG In total Allele A G	33 (27.3) 62 (51.7) 25 (20.8) 120 (100) 128 (53.3) 112 (46.7)	60 (27.3) 107 (48.6) 53 (24.1) 220 (100) 227 (51.6) 213 (48.4)	0.777 0.664	0.697

PitNET, pituitary adenoma; *p* – significance level when *p=*0.025.

Binary logistic regression also revealed no statistically significant differences between PitNET patients and the control group (**Table 6**).

**Table 6:** *MEG3* rs4081134 and rs7158663 binary logistic regression analysis within patients with PitNET and the control group.

Model	Genotype/Allele	OR (95% CI)	p-Value	AIC
*MEG3* rs4081134				
*Codominant*	AA vs. GG	1.076 (0.674-1.718)	0.760	445.250
	AG vs. GG	0.896 (0.407-1.973)	0.784	
*Dominant*	AA+AG vs. GG	1.040 (0.666-1.625)	0.862	443.459
*Recessive*	AA vs. GG+AG	0.864 (0.406-1.840)	0.705	443.334
*Overdominant*	AG vs. GG+AA	1.097 (0.701-1.717)	0.686	443.326
*Additive*	A	0.993 (0.708-1.393)	0.968	443.487
*MEG3* rs7158663				
*Codominant*	AG vs. GG	1.054 (0.622-1.785)	0.846	444.980
	AA vs. GG	0.858 (0.453-1.623)	0.637	
*Dominant*	GG+AG vs. AA	0.989 (0.600-1.628)	0.964	443.487
*Recessive*	GG vs. AA+AG	0.829 (0.484-1.420)	0.495	443.018
*Overdominant*	AG vs. AA+GG	1.129 (0.723-1.762)	0.593	443.204
*Additive*	G	0.933 (0.681-1.278)	0.664	443.301

OR, odds ratio; CI, confidence interval; p – significance level when p=0.025; AIC, Akaike information criterion.

The frequencies of genotypes and alleles for the selected SNVs were analyzed within the study groups, stratified by gender. However, there were no statistically significant differences in the distribution of genotypes and alleles and binary logistic regression analysis between females and males with PitNET and the control group (**Tables 7**–**9**).

**Table 7:** *MEG3* rs4081134 and rs7158663 genotype and allele frequencies in PitNET and control female groups.

Variant	PitNETgroup females, N (%)	Control group females, N (proc.)	*p-*Value
*MEG3* rs4081134 GG AG AA In total Allele G A	33 (47.8) 28 (40.6) 8 (11.6) 69 (100) 94 (68.1) 44 (31.9)	56 (46.7) 49 (40.8) 15 (12.5) 120 (100) 161 (67.1) 79 (32.9)	0.979 0.837
*MEG3* rs7158663 AA AG GG In total Allele A G	19 (27.5) 37 (53.6) 13 (18.9) 69 (100) 75 (54.4) 63 (45.6)	33 (27.5) 60 (50) 27 (22.5) 120 (100) 126 (52.5) 114 (47.5)	0.823 0.729

PitNET, pituitary adenoma; p – significance level when p=0.025.

**Table 8:** *MEG3* rs4081134 and rs7158663 genotype and allele frequencies in PitNET and control male groups.

Variant	PitNETgroup males, N (%)	Control group males, N (%)	*p-*Value
*MEG3* rs4081134 GG AG AA In total Allele G A	22 (43.1) 26 (51) 3 (5.9) 51 (100) 70 (68.6) 32 (31.4)	47 (47) 45 (45) 8 (8) 100 (100) 139 (69.5) 61 (30.5)	0.750 0.877
*MEG3* rs7158663 AA AG GG In total Allele A G	14 (27.5) 25 (49) 12 (23.5) 51 (100) 53 (52) 49 (48)	27 (27) 47 (47) 26 (26) 100 (100) 101 (50.5) 99 (49.5)	0.945 0.810

PitNET, pituitary adenoma; p – significance level when p=0.025.

**Table 9:** *MEG3* rs4081134 and rs7158663 binary logistic regression analysis within patients with PitNET and the control group, stratified by gender.

Model	Genotype/Allele	OR (95% CI)	p-Value	AIC
Males				
*MEG3* rs4081134				
Codominant	AA vs. GG	1.234 (0.613-2.485)	0.555	196.558
	AG vs. GG	0.801 (0.194-3.315)		
Dominant	AA+AG vs. GG	1.169 (0.593-2.305)	0.652	194.935
Recessive	AA vs. GG+AG	0.719 (0.182-2.834)	0.637	194.906
Overdominant	AG vs. GG+AA	1.271 (0.647-2.498)	0.487	194.654
Additive	A	1.047 (0.607-1.804)	0.870	195.111
*MEG3* rs7158663				
Codominant	AG vs. GG	1.026 (0.457-2.300)	0.951	197.024
	AA vs. GG	0.890 (0.348-2.280)	0.808	
Dominant	GG+AG vs. AA	0.977 (0.458-2.084)	0.953	195.135
Recessive	GG vs. AA+AG	0.876 (0.399-1.922)	0.741	195.028
Overdominant	AG vs. AA+GG	1.084 (0.552-2.130)	0.814	195.083
Additive	G	0.946 (0.593-1.508)	0.814	195.083
Females				
*MEG3* rs4081134				
Codominant	AA vs. GG	0.970 (0.515-1.826)	0.970	252.033
	AG vs. GG	0.905 (0.347-2.363)	0.347	
Dominant	AA+AG vs. GG	0.955 (0.528-1.727)	0.878	250.052
Recessive	AA vs. GG+AG	0.918 (0.368-2.290)	0.855	250.042
Overdominant	AG vs. GG+AA	0.990 (0.542-1.808)	0.973	250.074
Additive	A	0.957 (0.621-1.475)	0.842	250.036
*MEG3* rs7158663				
Codominant	AG vs. GG	1.071 (0.533-2.151)	0.847	251.682
	AA vs. GG	0.836 (0.350-1.995)	0.687	
Dominant	GG+AG vs. AA	0.998 (0.514-1.937)	0.996	250.076
Recessive	GG vs. AA+AG	0.800 (0.381-1.676)	0.554	249.720
Overdominant	AG vs. AA+GG	1.156 (0.639-2.093)	0.631	247.845
Additive	G	0.926 (0.604-1.419)	0.725	249.952

OR, odds ratio; CI, confidence interval; *p* – significance level when *p=*0.025; AIC, Akaike information criterion.

The frequencies of genotypes and alleles for the selected SNVs were also analyzed between microadenoma and macroadenoma and the control groups. However, there were no statistically significant differences in the distribution of genotypes and alleles and binary logistic regression analysis between microadenoma/macroadenoma and the control group (**Tables 10** and **11**).

**Table 10:** *MEG3* rs4081134 and rs7158663 genotype and allele frequencies in the control group and PitNET group, depending on the size of the PitNET.

Variant	Control group N (%) (N=220)	Microadenoma N (proc.) (N=43)	*p-*Value	MacroadenomaN (%) (N=77)	*p-*Value
*MEG3 rs4081134* GG AG AA In total Allele G A	103 (46.8) 94 (42.7) 23 (10.5) 220 (100) 300 (68.2) 140 (31.8)	22 (51.2) 19 (44.2) 2 (4.7) 43 (100) 63 (73.3) 23 (26.7)	0.488 0.352	33 (42.9) 35 (45.5) 9 (11.7) 77 (100) 101 (65.6) 53 (34.4)	0.830 0.553
*MEG3 rs7158663* AA AG GG In total Allele A G	60 (27.3) 107 (48.6) 53 (24.1) 220 (100) 227 (51.6) 213 (48.4)	11 (25.6) 26 (60.5) 6 (14) 43 (100) 48 (55.8) 38 (44.2)	0.262 0.473	22 (28.6) 36 (46.8) 19 (24.7) 77 (100) 80 (60) 74 (40)	0.958 0.939

*p* – significance level when *p=*0.025.

**Table 11:** *MEG3* rs4081134 and rs7158663 binary logistic regression analysis within patients with PitNET and the control group, stratified by PitNET size.

Model	Genotype/Allele	OR (95% CI)	p-Value	AIC
Microadenoma				
*MEG3* rs4081134				
Codominant	AA vs. GG	0.946 (0.482-1.858)	0.873	236.618
	AG vs. GG	0.407 (0.089-1.855)	0.245	
Dominant	AA+AG vs. GG	0.840 (0.437-1.616)	0.602	236.022
Recessive	AA vs. GG+AG	0.418 (0.095-1.842)	0.249	234.644
Overdominant	AG vs. GG+AA	1.061 (0.549-2.050)	0.860	236.263
Additive	A	0.781 (0.464-1.314)	0.352	235.404
*MEG3* rs7158663				
Codominant	AG vs. GG	1.325 (0.612-2.870)	0.475	235.449
	AA vs. GG	0.617 (0.214-1.784)	0.373	
Dominant	GG+AG vs. AA	1.091 (0.517-2.301)	0.819	236.241
Recessive	GG vs. AA+AG	0.511 (0.204-1.277)	0.151	233.972
Overdominant	AG vs. AA+GG	1.615 (0.830-3.144)	0.158	234.267
Additive	G	0.842 (0.527-1.345)	0.471	235.771
Macroadenoma				
*MEG3* rs4081134				
Codominant	AA vs. GG	1.162 (0.669-2.018)	0.593	343.561
	AG vs. GG	1.221 (0.514-2.900)	0.650	
Dominant	AA+AG vs. GG	1.174 (0.696-1.981)	0.548	341.573
Recessive	AA vs. GG+AG	1.134 (0.500-2.570)	0.764	341.846
Overdominant	AG vs. GG+AA	1.117 (0.663-1.883)	0.678	341.762
Additive	A	1.123 (0.763-1.653)	0.556	341.589
*MEG3* rs7158663				
Codominant	AG vs. GG	0.918 (0.495-1.701)	0.785	343.850
	AA vs. GG	0.978 (0.478-2.002)	0.951	
Dominant	GG+AG vs. AA	0.938 (0.527-1.669)	0.826	341.887
Recessive	GG vs. AA+AG	1.032 (0.565-1.887)	0.918	341.924
Overdominant	AG vs. AA+GG	0.927 (0.551-1.560)	0.776	341.854
Additive	G	0.986 (0.688-1.415)	0.940	341.929

OR, odds ratio; CI, confidence interval; *p* – significance level when *p=*0.025; AIC, Akaike information criterion.

The frequencies of genotypes and alleles for the selected SNVs were also analyzed between the PitNET and control group, depending on the recurrence, activeness, and invasiveness of the PitNET. However, there were no statistically significant differences in the distribution of genotypes and alleles and binary logistic regression analysis between the PitNET with recurrence, active, and invasive PitNET and the control groups (**Tables 12** and **13**).

**Table 12:** *MEG3* rs4081134 and rs7158663 genotype and allele frequencies in the control group and PitNET group, depending on the recurrence, activeness and invasiveness of the PitNET.

Variant	Control group N (%) (N=220)	PitNET with recurrence N (%) (N=23)	*p-*Value	Active PitNET N (%) (N=70)	*p-*Value	Invasive PitNET N (%) (N=56)	*p-*Value
*MEG3 rs4081134* GG AG AA In total Allele G A	103 (46.8) 94 (42.7) 23 (10.5) 220 (100) 300 (68.2) 140 (31.8)	11 (47.8) 10 (43.5) 2 (8.7) 23 (100) 32 (69.6) 14 (30.4)	0.966 0.847	30 (42.9) 31 (44.3) 9 (12.8) 70 (100) 91(65) 49(35)	0.783 0.484	24 (42.9) 26 (46.4) 6 (10.7) 56 (100) 74 (66.1) 38 (33.9)	0.863 0.670
*MEG3 rs7158663* AA AG GG In total Allele A G	60 (27.3) 107 (48.6) 53 (24.1) 220 (100) 227 (51.6) 213 (48.4)	7 (30.4) 8 (34.8) 8 (34.8) 23 (100) 22 (47.8) 24 (52.2)	0.393 0.626	22 (31.4) 34 (48.6) 14 (20) 70 (100) 78 (55.7) 62 (44.3)	0.701 0.394	13 (23.2) 31 (55.4) 12 (21.4) 56 (100) 57 (50.9) 55 (49.1)	0.665 0.895

PitNET– pituitary adenoma; *p* – significance level when *p=*0.025.

**Table 13:** *MEG3* rs4081134 and rs7158663 binary logistic regression analysis based on tumor recurrence, hormonal activity, and invasiveness.

Model	Genotype/Allele	OR (95% CI)	p-Value	AIC
PitNET with recurrence				
*MEG3* rs4081134				
Codominant	AA vs. GG	0.996 (0.405-2.452)	0.993	156.126
	AG vs. GG	0.814 (0.169-3.925)	0.798	
Dominant	AA+AG vs. GG	0.960 (0.406-2.269)	0.927	154.191
Recessive	AA vs. GG+AG	0.816 (0.180-3.705)	0.792	154.126
Overdominant	AG vs. GG+AA	1.031 (0.433-2.453)	0.945	154.194
Additive	A	0.938 (0.487-1.807)	0.849	154.162
*MEG3* rs7158663				
Codominant	AG vs. GG	0.641 (0.221-1.854)	0.412	154.347
	AA vs. GG	1.294 (0.440-3.808)	0.640	
Dominant	GG+AG vs. AA	0.857 (0.336-2.186)	0.747	154.097
Recessive	GG vs. AA+AG	1.681 (0.675-4.183)	0.265	153.012
Overdominant	AG vs. AA+GG	0.563 (0.229-1.382)	0.210	152.567
Additive	G	1.154 (0.638-2.088)	0.636	153.975
Active PitNET				
*MEG3* rs4081134				
Codominant	AA vs. GG	1.132 (0.637-2.011)	0.672	324.063
	AG vs. GG	1.343 (0.562-3.211)	0.507	
Dominant	AA+AG vs. GG	1.174 (0.682-2.019)	0.563	322.209
Recessive	AA vs. GG+AG	1.264 (0.555-2.876)	0.577	322.243
Overdominant	AG vs. GG+AA	1.065 (0.620-1.832)	0.819	322.493
Additive	A	1.151 (0.773-1.713)	0.489	322.069
*MEG3* rs7158663				
Codominant	AG vs. GG	0.867 (0.465-1.615)	0.867	323.831
	AA vs. GG	0.720 (0.335-1.548)	0.401	
Dominant	GG+AG vs. AA	0.818 (0.456-1.469)	0.502	322.100
Recessive	GG vs. AA+AG	0.788 (0.406-1.527)	0.480	322.034
Overdominant	AG vs. AA+GG	0.997 (0.582-1.708)	0.992	322.545
Additive	G	0.850 (0.583-1.241)	0.401	321.837
Invasive PitNET				
*MEG3* rs4081134				
Codominant	AA vs. GG	1.187 (0.638-2.209)	0.589	282.130
	AG vs. GG	1.120 (0.411-3.050)	0.825	
Dominant	AA+AG vs. GG	1.174 (0.649-2.121)	0.596	280.143
Recessive	AA vs. GG+AG	1.028 (0.397-2.569)	0.955	280.423
Overdominant	AG vs. GG+AA	1.162 (0.644-2.094)	0.618	280.178
Additive	A	1.100 (0.709-1.706)	0.671	280.246
*MEG3* rs7158663				
Codominant	AG vs. GG	1.337 (0.650-2.749)	0.429	281.608
	AA vs. GG	1.045 (0.439-2.487)	0.921	
Dominant	GG+AG vs. AA	1.240 (0.624-2.467)	0.539	280.040
Recessive	GG vs. AA+AG	0.859 (0.423-1.746)	0.675	280.247
Overdominant	AG vs. AA+GG	1.310 (0.726-2.361)	0.370	279.618
Additive	G	1.028 (0.679-1.558)	0.895	280.408

OR, odds ratio; CI, confidence interval; *p* – significance level when *p=*0.025; AIC, Akaike information criterion.

The frequencies of genotypes and alleles for the selected SNVs were also analyzed between the PitNET and control group, depending on the PitNET without recurrence, non-activeness, and non-invasiveness of the PitNET. However, there were no statistically significant differences in the distribution of genotypes and alleles and binary logistic regression analysis between the groups (**Tables 14** and **15**).

**Table 14:** *MEG3* rs4081134 and rs7158663 genotype and allele frequencies in control group and the PitNET group, depending on the PitNET without recurrence, non-activeness and non-invasiveness.

Variant	Control group N (%) (N=220)	PitNET without recurrence N (%) (N=97)	*p-*Value	Non-active PitNET N (%) (N=50)	*p-*Value	Non-active PitNET N (%) (N=64)	*p-*Value
*MEG3 rs4081134* GG AG AA In total Allele G A	103 (46.8) 94 (42.7) 23 (10.5) 220 (100) 300 (68.2) 140 (31.8)	44 (45.4) 44 (45.4) 9 (9.3) 97 (100) 132 (68) 62 (32)	0.891 0.972	25 (50) 23 (46) 2 (4) 50 (100) 73 (73) 27 (27)	0.364 0.347	31 (48.4) 28 (43.8) 5 (7.8) 64 (100) 90 (70.3) 38 (29.7)	0.823 0.647
*MEG3 rs7158663* AA AG GG In total Allele A G	60 (27.3) 107 (48.6) 53 (24.1) 220 (100) 227 (51.6) 213 (48.4)	26 (26.8) 54 (55.7) 17 (17.5) 97 (100) 106 (54.6) 88 (45.4)	0.372 0.479	11 (22) 28 (56) 11 (22) 50 (100) 50 (50) 50 (50)	0.623 0.774	20 (31.3) 31 (48.4) 13 (20.3) 64 (100) 71 (55.5) 57 (44.5)	0.747 0.439

PitNET, pituitary adenoma; *p* – significance level when *p=*0.025.

**Table 15:** *MEG3* rs4081134 and rs7158663 binary logistic regression analysis in non-recurrent, non-functioning, and non-invasive PitNET subgroups.

Model	Genotype/Allele	OR (95% CI)	p-Value	AIC
PitNET without recurrence				
*MEG3* rs4081134				
Codominant	AA vs. GG AG vs. GG	1.096 (0.663-1.812) 0.916 (0.392-2.138)	0.722 0.839	394.223
Dominant	AA+AG vs. GG	1.060 (0.656-1.713)	0.811	392.396
Recessive	AA vs. GG+AG	0.876 (0.389 -1.970)	0.749	392.350
Overdominant	AG vs. GG+AA	1.113 (0.688-1.800)	0.663	392.264
Additive	A	1.007 (0.700-1.447)	0.972	392.452
*MEG3* rs7158663				
Codominant	AG vs. GG AA vs. GG	1.165 (0.662-2.048) 0.740 (0.362-1.512)	0.597 0.409	392.431
Dominant	GG+AG vs. AA	1.024 (0.598-1.754)	0.931	392.446
Recessive	GG vs. AA+AG	0.670 (0.365-1.230)	0.196	390.713
Overdominant	AG vs. AA+GG	1.326 (0.821-2.143)	0.249	391.119
Additive	G	0.883 (0.627-1.243)	0.475	391.942
Non-active PitNET				
*MEG3* rs4081134				
Codominant	AA vs. GG AG vs. GG	1.008 (0.536-1.896) 0.358 (0.079-1.621)	0.980 0.183	260.337
Dominant	AA+AG vs. GG	0.880 (0.476-1.627)	0.684	260.584
Recessive	AA vs. GG+AG	0.357 (0.081-1.566)	0.172	258.337
Overdominant	AG vs. GG+AA	1.142 (0.616-2.116)	0.673	260.572
Additive	A	0.790 (0.484-1.288)	0.344	259.833
*MEG3* rs7158663				
Codominant	AG vs. GG AA vs. GG	1.427 (0.664-3.070) 1.132 (0.454-2.823)	0.362 0.790	261.793
Dominant	GG+AG vs. AA	1.330 (0.639-2.764)	0.446	260.147
Recessive	GG vs. AA+AG	0.889 (0.425-1.857)	0.754	260.650
Overdominant	AG vs. AA+GG	1.344 (0.725-2.493)	0.348	259.864
Additive	G	1.066 (0.690-1.636)	0.774	260.667
Non-invasive PitNET				
*MEG3* rs4081134				
Codominant	AA vs. GG AG vs. GG	0.990 (0.553-1.772) 0.772 (0.253-2.058)	0.972 0.722	306.674
Dominant	AA+AG vs. GG	0.937 (0.537-1.636)	0.819	305.032
Recessive	AA vs. GG+AG	0.726 (0.264-1.993)	0.534	304.676
Overdominant	AG vs. GG+AA	1.043 (0.595-1.828)	0.884	305.063
Additive	A	0.905 (0.589-1.389)	0.648	304.874
*MEG3* rs7158663				
Codominant	AG vs. GG AA vs. GG	0.869 (0.456-1.656) 0.736 (0.334-1.621)	0.670 0.447	306.498
Dominant	GG+AG vs. AA	0.825 (0.450-1.513)	0.534	304.702
Recessive	GG vs. AA+AG	0.803 (0.406-1.590)	0.529	304.678
Overdominant	AG vs. AA+GG	0.992 (0.568-1.732)	0.978	305.083
Additive	G	0.859 (0.581-1.270)	0.446	304.500

OR, odds ratio; CI, confidence interval; *p* – significance level when *p=*0.025; AIC, Akaike information criterion.

### 3.1 Immunohistochemistry

Immunohistochemical reactions using Ki-67, p53, and CK18 biomarkers were performed in 99 PitNET patients. Statistically significant differences were found when comparing clinical features of PitNET with the p53 H-score (**Table 16**). Macroadenomas were found to have a statistically significantly higher p53 H-score than microadenomas (median (IQR): 27 (29.65) vs. 16 (12.84), *p=*0.008, respectively). In addition, invasive PitNETs had a statistically significantly higher p53 H-score than non-invasive PitNETs (median (IQR): 27 (27.99) vs. 20 (19.31), *p=*0.018, respectively).

**Table 16:** Associations of clinical features of PitNET with p53 H-score.

PitNET group	p53 H-score median (IQR)	*p-*Value
Microadenoma	16 (12.84)	**0.008**
Macroadenoma	27 (29.65)	
PitNET with recurrence	18 (17.15)	0.075
PitNET without recurrence	26 (29.58)	
Active PitNET	26 (31.68)	0.373
Non-active PitNET	21 (2.16)	
Invasive PitNET	27 (27.99)	**0.018**
Non-invasive PitNET	20 (19.31)	

PitNET, pituitary adenoma; *p* – significance level when *p=*0.05; IQR, interquartile range.

p values in bold are statistically significant.

Comparison of the groups with different Ki-67 LI immunohistochemical reactions (<3% and >3%) revealed no statistically significant differences between the clinical PitNET groups (**Table 17**).

**Table 17:** Associations of clinical features of PitNET with Ki-67 LI.

PitNET group	Ki-67 labeling index n, (%)		*p-*Value
	<3%	>3%	
Microadenoma	20 (69)	9 (31)	0.555
Macroadenoma	42 (62.7)	25 (37.3)
Non-invasive PitNET	29 (67.4)	14 (32.6)	0.598
Invasive PitNET	33 (62.3)	20 (37.7)
PitNET without recurrence	46 (66.7)	23 (33.3)	0.495
PitNET with recurrence	16 (59.3)	11 (40.7)
Non-active PitNET	33 (67.3)	16 (32.7)	0.563
Active PitNET	29 (61.7)	18 (38.3)

PitNET, pituitary adenoma; *p* – significance level when *p=*0.05.

Statistically significant differences were found when comparing the groups of PitNET clinical features with the type of CK18 immunohistochemical reaction. Invasive PitNET was found to be characterized by negative CK18 reactions compared to the non-invasive PitNET group (24 (44.4%) vs. 6 (13.3%), *p* < 0.001, respectively). It was also found that non-active PitNETs had negative CK18 reactions compared to functionally active PitNETs (21 (42.0%) vs. 9 (18.4%), *p=*0.011, respectively). No statistically significant differences were found when comparing CK18 reactions between microadenoma and macroadenoma and between PitNET with or without recurrence (**Table 18**).

**Table 18:** Associations of clinical features of PitNET with CK18.

PitNET group	CK18 reaction, n (%)		*p-*Value
	Negative	Positive	
Microadenoma	9 (30)	21 (70)	0.965
Macroadenoma	21 (30.4)	48 (69.6)
Non-invasive PitNET	6 (13.3)	39 (86.7)	**<0.001**
Invasive PitNET	24 (44.4)	30 (55.6)
PitNET without recurrence	22 (31)	49 (69)	0.814
PitNET with recurrence	8 (28.6)	20 (71.4)
Non-active PitNET	21 (42)	29 (58)	**0.011**
Active PitNET	9 (18.4)	40 (81.6)

PitNET, pituitary adenoma; *p* – significance level when *p=*0.05.

p values in bold are statistically significant.

When comparing the distribution of *MEG3* rs4081134 and rs7158663 variants between negative and positive CK18 immunohistochemical reaction types, no statistically significant differences were observed (**Table 19**).

**Table 19:** *MEG3* rs4081134 and rs7158663 genotype and allele frequencies in CK18 negative and CK18 positive reaction groups.

Variant	Genotype/Allele	CK18 reaction, n (%)		*p-*Value
		Negative	Positive	
*MEG3* rs4081134	Genotype			0.303
	GG	7 (41.2)	20 (46.5)	
	AG	9 (52.9)	15 (34.9)	
	AA	1 (5.9)	8 (18.6)	
	In total	17 (100)	43 (100)	
	Allele			0.702
	G	23 (67.6)	\55 (64)	
	A	11 (32.4)	31 (36)	
*MEG3* rs7158663	Genotype			0.972
	AA	5 (29.4)	12 (27.9)	
	AG	7 (41.2)	17 (39.5)	
	GG	5 (29.4)	14 (32.6)	
	In total	17 (100)	43 (100)	
	Allele			0.818
	A	17 (50)	41 (47.7)	
	G	17 (50)	45 (52.3)	

*p* – significance level when *p=*0.025.

When comparing the distribution of *MEG3* rs4081134 and rs7158663 variants between the <3% and >3% Ki-67 LI in immunohistochemical reaction groups, no statistically significant differences were observed (**Table 20**).

**Table 20:** *MEG3* rs4081134 and rs7158663 genotype and allele frequencies in Ki-67 LI groups.

Variant	Genotype/Allele	Ki-67 labeling index, n (%)		*p-*Value
		<3%	>3%	
*MEG3* rs4081134	Genotype			0.319
	GG	18 (46.2)	9 (45)	
	AG	14 (35.9)	10 (50)	
	AA	7 (17.9)	1 (5)	
	In total	39 (100)	20 (100)	
	Allele			0.521
	G	50 (64.1)	28 (70)	
	A	28 (35.9)	12 (30)	
*MEG3* rs7158663	Genotype			0.441
	AA	10 (25.6)	7 (35)	
	AG	15 (38.5)	9 (45)	
	GG	14 (35.9)	4 (20)	
	In total	39 (100)	20 (100)	
	Allele			0.194
	A	35 (44.9)	23 (57.5)	
	G	43 (55.1)	17 (42.5)	

*p* – significance level when *p=*0.025.

**Figure 1:**
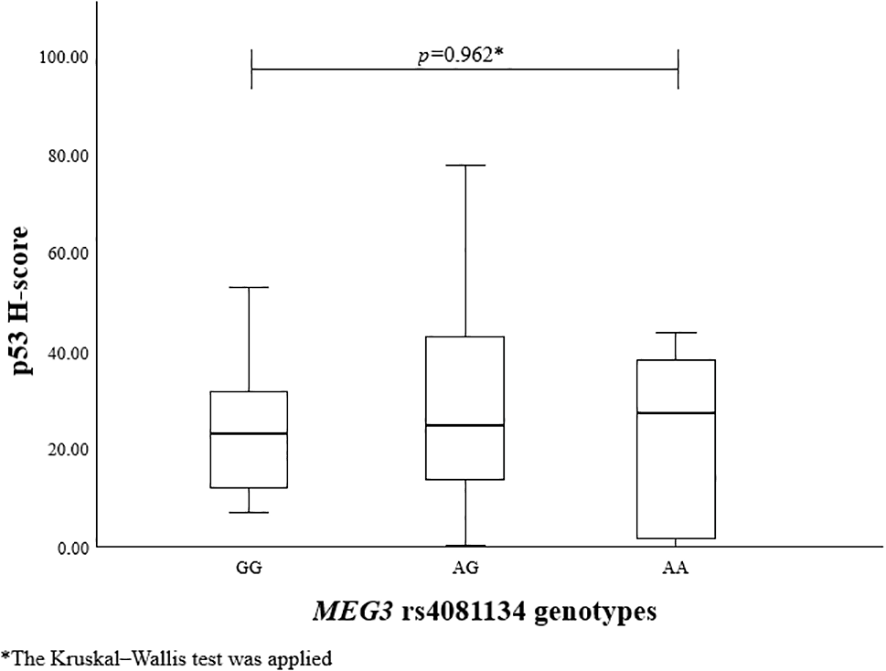
p53 H-score in different *MEG3* rs4081134 genotype groups.

**Figure 2:**
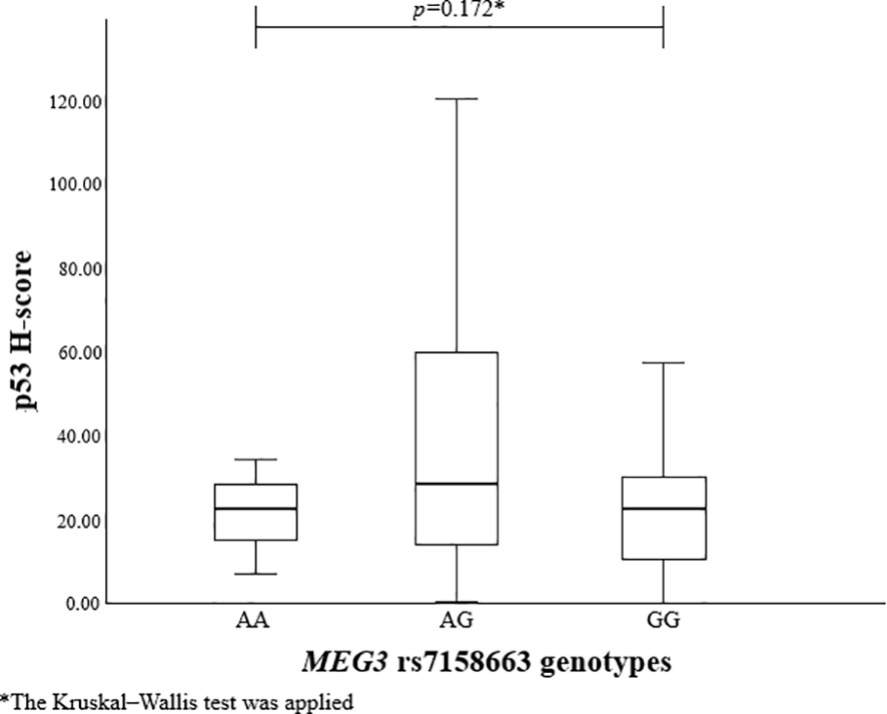
p53 H-score in different *MEG3* rs7158663 genotype groups.

To evaluate the association between *MEG3* rs4081134 and rs7158663 variants and p53, the p53 H-score was calculated in different genotype groups. However, no statistically significant differences were observed (**Figures 1** and **2**).

## 4 Discussion

Tumor biomarkers have been proven to play a crucial role in cancer screening and early diagnosis, prognosis prediction, monitoring for recurrence, and evaluating treatment response. Currently, the ongoing research for novel, highly sensitive and specific biomarkers has been a cornerstone of personalized medicine and has dramatically improved outcomes for cancer patients (35).

Our study investigated the relationships between *MEG3* rs7158663 and rs4081134 variants, Ki-67 LI, p53 H-score, and CK18 immunohistochemical reactions with the clinicopathological features of PAs. *MEG3* is an lncRNA mostly known to be involved in the regulation of *p53* tumor suppressor gene expression. In recent years, both *MEG3* and its genetic variants have received significant attention within the scientific field due to their potential involvement in tumorigenesis. Interestingly, the *MEG3* rs7158663 variant is found to be associated with increased susceptibility to lung, colorectal, and gastric cancers. According to Wang and co-authors, *MEG3* rs7158663 variant may disrupt the binding of specific miRNAs, such as hsa-miR-4307 and hsa-miR-1265, to *MEG3*. Such interference could impact gene regulation mechanisms and potentially contribute to cancer development (36). While these associations have been well-documented in multiple malignancies, it is important to highlight that the role of *MEG3* and its variants, particularly rs7158663, has not been extensively studied in the context of PitNETs. Although the general function of *MEG3* in tumor suppression is being gradually uncovered, its involvement in PitNET pathophysiology remains relatively unexplored. The study by Wang and co-authors showed that low *MEG3* expression was significantly associated with larger PitNET size (>3 cm), increased invasiveness and advanced clinical stage (III-IV). Moreover, *MEG3* overexpression was related to inhibition of pituitary cell proliferation and induction of apoptosis (37). A study conducted by Tang and colleagues demonstrated the tumor-suppressive role of *MEG3* lncRNA, showing that mutations in the α subunit of the stimulatory G protein (GNAS) upregulate *MEG3* lncRNA expression and are associated with less invasive growth hormone-secreting PitNETs (38). In our study, neither the *MEG3* rs7158663 nor rs4081134 variants showed any significant associations with PitNETs. Furthermore, no associations were observed between the analyzed variants and Ki-67 LI, p53 H-score, or CK18 immunohistochemical reactions. These findings suggest that, despite the established relevance of *MEG3* variants in other cancers, their role in PitNETs remains unclear and warrants further investigation.

Ki-67 is another biomarker that is widely studied across different types of cancer. According to the 2022 WHO Classification of Neuroendocrine Tumors, the Ki-67 LI is no longer used as a formal diagnostic criterion for PitNETs, despite being recognized as a biomarker of aggressive tumor behavior (7). However, a study by Bălinişteanu and colleagues demonstrated a significant positive correlation between prolactin expression and the Ki-67 LI. According to the researchers, no other pituitary hormone showed a statistically significant association with proliferation rate. Moreover, high Ki-67 LI (>3%) was linked to more aggressive tumor behavior, warranting closer radiological and endocrinological follow-up (39). Grimm and co-authors revealed that the Ki-67 proliferation index does not predict invasiveness or the size of PAs. In addition, the Ki-67 LI did not show any significant associations with cavernous-sine invasion (Knosp grade) (40). Conversely, a study by Yuhan and co-authors demonstrated that higher Ki-67 indices of functional PitNETs corresponded to lower Knosp grades. On the other hand, higher Ki-67 indices of nonfunctional PitNETs were associated with higher Knosp grades, indicating that nonfunctional PitNETs can grow larger and more invasive before diagnosis, allowing proliferative tumors to extend into the cavernous sinus (41). However, a recently published study emphasized that Ki-67 LI results are highly dependent on the specific assessment method used. Accordingly, Loughrey et al. proved that digital Ki-67 LI was significantly higher when scored on 1,000 cells vs. 10,000 cells, leading to a substantial margin of error when Ki-67 LI is being assessed on a lower number of cells (42). In our study, we employed a different methodology by using the hot-spot counting method to assess the Ki-67 LI, which is designed to target the most proliferative and potentially aggressive areas within the tumor. Despite this focused approach, we took a different approach in the assessment of the Ki-67 LI by using the hot-spot method, which is thought to represent the most aggressive clones within a tumor. However, no statistically significant associations were found between Ki-67 LI (<3% vs. >3%) groups and clinicopathological characteristics of PitNETs (tumor size, invasiveness, recurrence, functional activity). This lack of correlation may reflect the biological heterogeneity of PitNETs, where proliferation markers like Ki-67 alone might not sufficiently capture the complex mechanisms driving tumor behavior. Alternatively, it may suggest that Ki-67 LI, even when assessed by hot-spot analysis, has limited prognostic value when used in isolation. We also acknowledge that our Ki-67 assessment did not meet the recommended standard of 1,000–5,000 cells per sample. To address this limitation, we analyzed a relatively large cohort (99 cases) and restricted counts to 300 cells in the most proliferative regions. While this approach may slightly overestimate the proliferative fraction, it provided consistent manual evaluation across all samples.

p53 is a transcription factor responsible for modulating various stress-induced antiproliferative pathways. Despite being widely studied in different malignancies, its role in the PitNETs remains unclear (43). Wang et al. revealed that miR-219a-2-3p inhibits PitNET cell proliferation, and promotes apoptosis by modulating the *MDM2*/*p53* axis. Accordingly, miR-219a-2-3p was significantly down-regulated in PitNET cells. However, artificial overexpression of miR-219a-2-3p inhibited PitNET cell proliferation and promoted apoptosis and p53 expression (44). Abnormal p53 expression has also been reported in poorly differentiated neuroendocrine carcinomas (NECs), correlating with aggressive behavior and poorer prognosis, whereas well-differentiated NETs typically retain normal p53 expression (45, 46). In our study, we found that a higher p53 H-score was associated with invasive PitNETs, indicating that elevated p53 expression may contribute to PitNET invasiveness. Additionally, we found that a higher p53 H-score was significantly associated with larger tumor size, showing that increased p53 expression may contribute to clinical symptoms related to tumor compression. These findings suggest that p53 immunohistochemical scoring could serve as a useful tool for risk evaluation in PitNET patients. Incorporating p53 H-score into routine clinical practice may help identify tumors at higher risk of invasiveness and aggressive growth.

To date, the relationship between CK18 immunostaining patterns and the clinicopathological features of PitNETs has not yet been established. Although, CK18 expression has been extensively studied in various other malignancies, where it is often associated with tumor differentiation, proliferative activity, and overall prognosis. Yang et al. highlighted that elevated serum CK18 levels are associated with poorer breast cancer prognosis, whereas high CK18 tissue expression indicates a better outcome. Additionally, high CK18 expression has been found to be associated with larger tumor size, stage, and grade (47). Shi and co-authors revealed that CK18 expression is suppressed in advanced and metastatic breast tumors. Moreover, CK18 loss was found to activate the NF-κB/Snail axis, leading to the upregulation of breast cancer resistance protein (BCRP) (48). Safadi et al. demonstrated that CK18 expression rises with tumor aggressiveness. Stronger CK18 immunohistochemical staining scores were significantly associated with advanced clinical stage and greater invasion of oral and oropharyngeal squamous cell carcinoma. Besides, elevated CK18 expression showed higher chances of tumor recurrence after surgical resection (49). A study by Yin et al. showed that CK18 expression in prostate cancer inversely correlates with tumor grade. Lower CK18 staining intensity was associated with higher Gleason scores (>7), indicating CK18 downregulation in aggressive prostate tumors (50). In our study, we found that invasive PitNETs are associated with negative CK18 reactions compared to the non-invasive PitNET group, showing that loss of CK18 expression may be related to PitNET tumor aggressiveness. Also, an additional consideration is the heterogeneity of CK18 staining patterns observed in our study, including diffuse, focal, cytoplasmic, perinuclear, fibrous body, and ring-like perinuclear reactions. Such variability may reflect underlying biological differences in tumor architecture or cell stress responses, and future studies are warranted to clarify whether distinct patterns (e.g., fibrous body vs. diffuse) carry prognostic or diagnostic significance. Additionally, we found that functionally inactive PitNETs are more likely to demonstrate negative CK18 reactions compared to functionally active PitNET groups, indicating that CK18 downregulation may be linked to the loss of hormonal production. As with p53, these findings suggest that CK18 immunostaining could serve as a practical biomarker in PitNETs, helping to predict tumor invasiveness and functional status.

## 5 Conclusion

This study found that elevated p53 H-score was significantly associated with both larger PitNET size and invasive behavior. Assuming that a higher p53 H-score can be associated with more aggressive progression of PitNET. Additionally, negative CK18 staining was significantly associated with non-functioning PitNETs and invasiveness. This result suggests that the absence of CK18 expression may indicate a more aggressive tumor phenotype and reduced hormonal activity. These findings indicate that p53 expression level and CK18 status may serve as a useful prognostic tool in PitNETs.

## 6 Limitations

We assessed the Ki-67 labeling index by counting 300 cells in hotspot regions across a relatively large cohort of 99 cases, ensuring consistent evaluation. Future studies with larger cohorts and automated methods may further enhance the robustness of these findings.

## Data Availability

The original contributions presented in the study are included in the article/**Supplementary Material**. The datasets of genotype analysis are available in the European Variation Archive repository (Project number: PRJEB96895, Analyses: HGV analysis=>ERZ28422108). Direct link: https://www.ebi.ac.uk/eva/?eva-study=PRJEB96895. The immunohistochemical data are provided in Figshare: https://doi.org/10.6084/m9.figshare.30000169.v1.
