# Supplementary material for: Associations of MEG3 rs7158663, rs4081134 gene variants and Ki-67, p53, CK18 immunohistochemical markers with clinical features of pituitary neuroendocrine tumors
Source: Front Endocrinol (Lausanne). 2025 Oct 9;16:1657520. doi: 10.3389/fendo.2025.1657520 (PMC12545000; doi:10.3389/fendo.2025.1657520)
Supplement: Supplementary file 1 [file Table1.docx]

Supplementary material

***Table S1. MEG3 rs4081134 and rs7158663 binary logistic regression analysis within patients with PitNET and control group***

| Model | Genotype/Allele | OR (95% CI) | *p-*Value | AIC |
| --- | --- | --- | --- | --- |
| **MEG3 rs4081134** | | | | |
| Codominant | AA *vs.* GG  AG *vs.* GG | 1.076 (0.674-1.718)  0.896 (0.407-1.973) | 0.760  0.784 | 445.250 |
| Dominant | AA+AG *vs.* GG | 1.040 (0.666-1.625) | 0.862 | 443.459 |
| Recessive | AA *vs.* GG+AG | 0.864 (0.406-1.840) | 0.705 | 443.334 |
| Overdominant | AG *vs*. GG+AA | 1.097 (0.701-1.717) | 0.686 | 443.326 |
| Additive | A | 0.993 (0.708-1.393) | 0.968 | 443.487 |
| **MEG3 rs7158663** | | | | |
| Codominant | AG *vs.* GG  AA *vs.* GG | 1.054 (0.622-1.785)  0.858 (0.453-1.623) | 0.846  0.637 | 444.980 |
| Dominant | GG+AG *vs.* AA | 0.989 (0.600-1.628) | 0.964 | 443.487 |
| Recessive | GG *vs.* AA+AG | 0.829 (0.484-1.420) | 0.495 | 443.018 |
| Overdominant | AG *vs*. AA+GG | 1.129 (0.723-1.762) | 0.593 | 443.204 |
| Additive | G | 0.933 (0.681-1.278) | 0.664 | 443.301 |

OR – odds ratio; CI – confidence interval; *p* – significance level when *p* = 0.025; AIC – Akaike information criterion.

***Table S2. MEG3 rs4081134 and rs7158663 binary logistic regression analysis within patients with PitNET and control group, stratified by gender***

| Model | Genotype/Allele | OR (95% CI) | *p-*Value | AIC |  |
| --- | --- | --- | --- | --- | --- |
| Males | | | | |  |
| **MEG3 rs4081134** | | | | |  |
| Codominant | AA *vs.* GG  AG *vs.* GG | 1.234 (0.613-2.485)  0.801 (0.194-3.315) | 0.555 | 196.558 |  |
| Dominant | AA+AG *vs.* GG | 1.169 (0.593-2.305) | 0.652 | 194.935 |  |
| Recessive | AA *vs.* GG+AG | 0.719 (0.182-2.834) | 0.637 | 194.906 |  |
| Overdominant | AG *vs*. GG+AA | 1.271 (0.647-2.498) | 0.487 | 194.654 |  |
| Additive | A | 1.047 (0.607-1.804) | 0.870 | 195.111 |  |
| **MEG3 rs7158663** | | | | |  |
| Codominant | AG *vs.* GG  AA *vs.* GG | 1.026 (0.457-2.300)  0.890 (0.348-2.280) | 0.951  0.808 | 197.024 |  |
| Dominant | GG+AG *vs.* AA | 0.977 (0.458-2.084) | 0.953 | 195.135 |  |
| Recessive | GG *vs.* AA+AG | 0.876 (0.399-1.922) | 0.741 | 195.028 |  |
| Overdominant | AG *vs*. AA+GG | 1.084 (0.552-2.130) | 0.814 | 195.083 |  |
| Additive | G | 0.946 (0.593-1.508) | 0.814 | 195.083 |  |
| Females | | | | | |
| **MEG3 rs4081134** | | | | | |
| Codominant | | AA *vs.* GG  AG *vs.* GG | 0.970 (0.515-1.826)  0.905 (0.347-2.363) | 0.970  0.347 | 252.033 |
| Dominant | | AA+AG *vs.* GG | 0.955 (0.528-1.727) | 0.878 | 250.052 |
| Recessive | | AA *vs.* GG+AG | 0.918 (0.368-2.290) | 0.855 | 250.042 |
| Overdominant | | AG *vs*. GG+AA | 0.990 (0.542-1.808) | 0.973 | 250.074 |
| Additive | | A | 0.957 (0.621-1.475) | 0.842 | 250.036 |
| **MEG3 rs7158663** | | | | | |
| Codominant | | AG *vs.* GG  AA *vs.* GG | 1.071 (0.533-2.151)  0.836 (0.350-1.995) | 0.847  0.687 | 251.682 |
| Dominant | | GG+AG *vs.* AA | 0.998 (0.514-1.937) | 0.996 | 250.076 |
| Recessive | | GG *vs.* AA+AG | 0.800 (0.381-1.676) | 0.554 | 249.720 |
| Overdominant | | AG *vs*. AA+GG | 1.156 (0.639-2.093) | 0.631 | 247.845 |
| Additive | | G | 0.926 (0.604-1.419) | 0.725 | 249.952 |

OR – odds ratio; CI – confidence interval; *p* – significance level when *p* = 0.025; AIC – Akaike information criterion.

***Table S3. MEG3 rs4081134 and rs7158663 binary logistic regression analysis within patients with PitNET and control group, stratified by tumor size***

| Model | Genotype/Allele | OR (95% CI) | *p-*Value | AIC |  |
| --- | --- | --- | --- | --- | --- |
| Microadenoma | | | | |  |
| **MEG3 rs4081134** | | | | |  |
| Codominant | AA *vs.* GG  AG *vs.* GG | 0.946 (0.482-1.858)  0.407 (0.089-1.855) | 0.873  0.245 | 236.618 |  |
| Dominant | AA+AG *vs.* GG | 0.840 (0.437-1.616) | 0.602 | 236.022 |  |
| Recessive | AA *vs.* GG+AG | 0.418 (0.095-1.842) | 0.249 | 234.644 |  |
| Overdominant | AG *vs*. GG+AA | 1.061 (0.549-2.050) | 0.860 | 236.263 |  |
| Additive | A | 0.781 (0.464-1.314) | 0.352 | 235.404 |  |
| **MEG3 rs7158663** | | | | |  |
| Codominant | AG *vs.* GG  AA *vs.* GG | 1.325 (0.612-2.870)  0.617 (0.214-1.784) | 0.475  0.373 | 235.449 |  |
| Dominant | GG+AG *vs.* AA | 1.091 (0.517-2.301) | 0.819 | 236.241 |  |
| Recessive | GG *vs.* AA+AG | 0.511 (0.204-1.277) | 0.151 | 233.972 |  |
| Overdominant | AG *vs*. AA+GG | 1.615 (0.830-3.144) | 0.158 | 234.267 |  |
| Additive | G | 0.842 (0.527-1.345) | 0.471 | 235.771 |  |
| Macroadenoma | | | | | |
| **MEG3 rs4081134** | | | | | |
| Codominant | | AA *vs.* GG  AG *vs.* GG | 1.162 (0.669-2.018)  1.221 (0.514-2.900) | 0.593  0.650 | 343.561 |
| Dominant | | AA+AG *vs.* GG | 1.174 (0.696-1.981) | 0.548 | 341.573 |
| Recessive | | AA *vs.* GG+AG | 1.134 (0.500-2.570) | 0.764 | 341.846 |
| Overdominant | | AG *vs*. GG+AA | 1.117 (0.663-1.883) | 0.678 | 341.762 |
| Additive | | A | 1.123 (0.763-1.653) | 0.556 | 341.589 |
| **MEG3 rs7158663** | | | | | |
| Codominant | | AG *vs.* GG  AA *vs.* GG | 0.918 (0.495-1.701)  0.978 (0.478-2.002) | 0.785  0.951 | 343.850 |
| Dominant | | GG+AG *vs.* AA | 0.938 (0.527-1.669) | 0.826 | 341.887 |
| Recessive | | GG *vs.* AA+AG | 1.032 (0.565-1.887) | 0.918 | 341.924 |
| Overdominant | | AG *vs*. AA+GG | 0.927 (0.551-1.560) | 0.776 | 341.854 |
| Additive | | G | 0.986 (0.688-1.415) | 0.940 | 341.929 |

OR – odds ratio; CI – confidence interval; *p* – significance level when *p* = 0.025;

AIC – Akaike information criterion.

***Table S4. MEG3 rs4081134 and rs7158663 binary logistic regression analysis based on tumor recurrence, hormonal activity, and invasiveness***

| Model | Genotype/Allele | OR (95% CI) | *p-*Value | AIC |  |
| --- | --- | --- | --- | --- | --- |
| PitNET with recurrence | | | | |  |
| **MEG3 rs4081134** | | | | |  |
| Codominant | AA *vs.* GG  AG *vs.* GG | 0.996 (0.405-2.452)  0.814 (0.169-3.925) | 0.993  0.798 | 156.126 |  |
| Dominant | AA+AG *vs.* GG | 0.960 (0.406-2.269) | 0.927 | 154.191 |  |
| Recessive | AA *vs.* GG+AG | 0.816 (0.180-3.705) | 0.792 | 154.126 |  |
| Overdominant | AG *vs*. GG+AA | 1.031 (0.433-2.453) | 0.945 | 154.194 |  |
| Additive | A | 0.938 (0.487-1.807) | 0.849 | 154.162 |  |
| **MEG3 rs7158663** | | | | |  |
| Codominant | AG *vs.* GG  AA *vs.* GG | 0.641 (0.221-1.854)  1.294 (0.440-3.808) | 0.412  0.640 | 154.347 |  |
| Dominant | GG+AG *vs.* AA | 0.857 (0.336-2.186) | 0.747 | 154.097 |  |
| Recessive | GG *vs.* AA+AG | 1.681 (0.675-4.183) | 0.265 | 153.012 |  |
| Overdominant | AG *vs*. AA+GG | 0.563 (0.229-1.382) | 0.210 | 152.567 |  |
| Additive | G | 1.154 (0.638-2.088) | 0.636 | 153.975 |  |
| Active PitNET | | | | | |
| **MEG3 rs4081134** | | | | | |
| Codominant | | AA *vs.* GG  AG *vs.* GG | 1.132 (0.637-2.011)  1.343 (0.562-3.211) | 0.672  0.507 | 324.063 |
| Dominant | | AA+AG *vs.* GG | 1.174 (0.682-2.019) | 0.563 | 322.209 |
| Recessive | | AA *vs.* GG+AG | 1.264 (0.555-2.876) | 0.577 | 322.243 |
| Overdominant | | AG *vs*. GG+AA | 1.065 (0.620-1.832) | 0.819 | 322.493 |
| Additive | | A | 1.151 (0.773-1.713) | 0.489 | 322.069 |
| **MEG3 rs7158663** | | | | | |
| Codominant | | AG *vs.* GG  AA *vs.* GG | 0.867 (0.465-1.615)  0.720 (0.335-1.548) | 0.867  0.401 | 323.831 |
| Dominant | | GG+AG *vs.* AA | 0.818 (0.456-1.469) | 0.502 | 322.100 |
| Recessive | | GG *vs.* AA+AG | 0.788 (0.406-1.527) | 0.480 | 322.034 |
| Overdominant | | AG *vs*. AA+GG | 0.997 (0.582-1.708) | 0.992 | 322.545 |
| Additive | | G | 0.850 (0.583-1.241) | 0.401 | 321.837 |
| Invasive PitNET | | | | | |
| MEG3 rs4081134 | | | | | |
| Codominant | | AA *vs.* GG  AG *vs.* GG | 1.187 (0.638-2.209)  1.120 (0.411-3.050) | 0.589  0.825 | 282.130 |
| Dominant | | AA+AG *vs.* GG | 1.174 (0.649-2.121) | 0.596 | 280.143 |
| Recessive | | AA *vs.* GG+AG | 1.028 (0.397-2.569) | 0.955 | 280.423 |
| Overdominant | | AG *vs*. GG+AA | 1.162 (0.644-2.094) | 0.618 | 280.178 |
| Additive | | A | 1.100 (0.709-1.706) | 0.671 | 280.246 |
| MEG3 rs7158663 | | | | | |
| Codominant | | AG *vs.* GG  AA *vs.* GG | 1.337 (0.650-2.749)  1.045 (0.439-2.487) | 0.429  0.921 | 281.608 |
| Dominant | | GG+AG *vs.* AA | 1.240 (0.624-2.467) | 0.539 | 280.040 |
| Recessive | | GG *vs.* AA+AG | 0.859 (0.423-1.746) | 0.675 | 280.247 |
| Overdominant | | AG *vs*. AA+GG | 1.310 (0.726-2.361) | 0.370 | 279.618 |
| Additive | | G | 1.028 (0.679-1.558) | 0.895 | 280.408 |

PitNET – pituitary neuroendocrine tumor; OR – odds ratio; CI – confidence interval; *p* – significance level when *p* = 0.025; AIC – Akaike information criterion.

***Table S5. MEG3 rs4081134 and rs7158663 binary logistic regression analysis in non-recurrent, non-functioning, and non-invasive PitNET subgroups***

| Model | Genotype/Allele | OR (95% CI) | *p-*Value | AIC |
| --- | --- | --- | --- | --- |
| PitNET without recurrence | | | | |
| **MEG3 rs4081134** | | | | |
| Codominant | AA *vs.* GG  AG *vs.* GG | 1.096 (0.663-1.812)  0.916 (0.392-2.138) | 0.722  0.839 | 394.223 |
| Dominant | AA+AG *vs.* GG | 1.060 (0.656-1.713) | 0.811 | 392.396 |
| Recessive | AA *vs.* GG+AG | 0.876 (0.389 -1.970) | 0.749 | 392.350 |
| Overdominant | AG *vs*. GG+AA | 1.113 (0.688-1.800) | 0.663 | 392.264 |
| Additive | A | 1.007 (0.700-1.447) | 0.972 | 392.452 |
| **MEG3 rs7158663** | | | | |
| Codominant | AG *vs.* GG  AA *vs.* GG | 1.165 (0.662-2.048)  0.740 (0.362-1.512) | 0.597  0.409 | 392.431 |
| Dominant | GG+AG *vs.* AA | 1.024 (0.598-1.754) | 0.931 | 392.446 |
| Recessive | GG *vs.* AA+AG | 0.670 (0.365-1.230) | 0.196 | 390.713 |
| Overdominant | AG *vs*. AA+GG | 1.326 (0.821-2.143) | 0.249 | 391.119 |
| Additive | G | 0.883 (0.627-1.243) | 0.475 | 391.942 |
| Non-active PitNET | | | | |
| **MEG3 rs4081134** | | | | |
| Codominant | AA *vs.* GG  AG *vs.* GG | 1.008 (0.536-1.896)  0.358 (0.079-1.621) | 0.980  0.183 | 260.337 |
| Dominant | AA+AG *vs.* GG | 0.880 (0.476-1.627) | 0.684 | 260.584 |
| Recessive | AA *vs.* GG+AG | 0.357 (0.081-1.566) | 0.172 | 258.337 |
| Overdominant | AG *vs*. GG+AA | 1.142 (0.616-2.116) | 0.673 | 260.572 |
| Additive | A | 0.790 (0.484-1.288) | 0.344 | 259.833 |
| **MEG3 rs7158663** | | | | |
| Codominant | AG *vs.* GG  AA *vs.* GG | 1.427 (0.664-3.070)  1.132 (0.454-2.823) | 0.362  0.790 | 261.793 |
| Dominant | GG+AG *vs.* AA | 1.330 (0.639-2.764) | 0.446 | 260.147 |
| Recessive | GG *vs.* AA+AG | 0.889 (0.425-1.857) | 0.754 | 260.650 |
| Overdominant | AG *vs*. AA+GG | 1.344 (0.725-2.493) | 0.348 | 259.864 |
| Additive | G | 1.066 (0.690-1.636) | 0.774 | 260.667 |
| Non-invasive PitNET | | | | |
| MEG3 rs4081134 | | | | |
| Codominant | AA *vs.* GG  AG *vs.* GG | 0.990 (0.553-1.772)  0.772 (0.253-2.058) | 0.972  0.722 | 306.674 |
| Dominant | AA+AG *vs.* GG | 0.937 (0.537-1.636) | 0.819 | 305.032 |
| Recessive | AA *vs.* GG+AG | 0.726 (0.264-1.993) | 0.534 | 304.676 |
| Overdominant | AG *vs*. GG+AA | 1.043 (0.595-1.828) | 0.884 | 305.063 |
| Additive | A | 0.905 (0.589-1.389) | 0.648 | 304.874 |
| MEG3 rs7158663 | | | | |
| Codominant | AG *vs.* GG  AA *vs.* GG | 0.869 (0.456-1.656)  0.736 (0.334-1.621) | 0.670  0.447 | 306.498 |
| Dominant | GG+AG *vs.* AA | 0.825 (0.450-1.513) | 0.534 | 304.702 |
| Recessive | GG *vs.* AA+AG | 0.803 (0.406-1.590) | 0.529 | 304.678 |
| Overdominant | AG *vs*. AA+GG | 0.992 (0.568-1.732) | 0.978 | 305.083 |
| Additive | G | 0.859 (0.581-1.270) | 0.446 | 304.500 |

PitNET – pituitary neuroendocrine tumor; OR – odds ratio; CI – confidence interval; *p* – significance level when *p* = 0.025; AIC – Akaike information criterion.

***Table S6. Associations of clinical features of PitNET with Ki-67 LI***

| **PitNET group** | **Ki-67 labeling index n, (%)** | | ***p-*Value** |
| --- | --- | --- | --- |
|  | **<3%** | **>3%** |  |
| Microadenoma | 20 (69) | 9 (31) | 0.555 |
| Macroadenoma | 42 (62.7) | 25 (37.3) |  |
| Non-invasive | 29 (67.4) | 14 (32.6) | 0.598 |
| Invasive | 33 (62.3) | 20 (37.7) |  |
| PitNET without recurrence | 46 (66.7) | 23 (33.3) | 0.495 |
| PitNET with recurrence | 16 (59.3) | 11 (40.7) |  |
| Non-active | 33 (67.3) | 16 (32.7) | 0.563 |
| Active | 29 (61.7) | 18 (38.3) |  |

PitNET – pituitary neuroendocrine tumor; *p* – significance level when *p* = 0.05

***Table S7. MEG3 rs4081134 and rs7158663 genotype and allele frequencies in CK18 negative and CK18 positive reaction groups***

| **Variant** | **Genotype/Allele** | **CK18 reaction, n (%)** | | ***p-*Value** |
| --- | --- | --- | --- | --- |
|  |  | **Negative** | **Positive** |  |
| ***MEG3* rs4081134** | Genotype  GG  AG  AA  Viso | 7 (41.2)  9 (52.9)  1 (5.9)  17 (100) | 20 (46.5)  15 (34.9)  8 (18.6)  43 (100) | 0.303 |
|  | Allele  G  A | 23 (67.6)  11 (32.4) | 55 (64)  31 (36) | 0.702 |
| ***MEG3* rs7158663** | Genotype  AA  AG  GG  Viso | 5 (29.4)  7 (41.2)  5 (29.4)  17 (100) | 12 (27.9)  17 (39.5)  14 (32.6)  43 (100) | 0.972 |
|  | Allele  A  G | 17 (50)  17 (50) | 41 (47.7)  45 (52.3) | 0.818 |

*p* – significance level when *p* = 0.025.

***Table S8. MEG3 rs4081134 and rs7158663 genotype and allele frequencies in Ki-67 LI groups***

| **Variant** | **Genotype/Allele** | **Ki-67 labeling index, n (%)** | | ***p-*Value** |
| --- | --- | --- | --- | --- |
|  |  | **<3%** | **>3%** |  |
| ***MEG3* rs4081134** | Genotype  GG  AG  AA  In total | 18 (46.2)  14 (35.9)  7 (17.9)  39 (100) | 9 (45)  10 (50)  1 (5)  20 (100) | 0.319 |
|  | Allele  G  A | 50 (64.1)  28 (35.9) | 28 (70)  12 (30) | 0.521 |
| ***MEG3* rs7158663** | Genotype  AA  AG  GG  In total | 10 (25.6)  15 (38.5)  14 (35.9)  39 (100) | 7 (35)  9 (45)  4 (20)  20 (100) | 0.441 |
|  | Allele  A  G | 35 (44.9)  43 (55.1) | 23 (57.5)  17 (42.5) | 0.194 |

*p* – significance level when *p* = 0.025
